# A Facile Flow-Casting Production of Bioactive Glass Coatings on Porous Titanium for Bone Tissue Engineering

**DOI:** 10.3390/ma11091540

**Published:** 2018-08-27

**Authors:** Haiou Yang, Qijie Zhu, Hongfei Qi, Xianhu Liu, Meixia Ma, Qiang Chen

**Affiliations:** 1 State Key Laboratory of Solidification Processing, Northwestern Polytechnical University, Xi’an 710072, China; yanghaiou@nwpu.edu.cn (H.Y.); mameixia66@foxmail.com (M.M.); 2 Key Laboratory for Space Bioscience and Biotechnology, School of Life Sciences, Northwestern Polytechnical University, Xi’an 710072, China; zhuqijietsc@outlook.com (Q.Z.); qihongfei@mail.nwpu.edu.cn (H.Q.); 3 National Engineering Research Center for Advanced Polymer Processing Technology, Zhengzhou University, Zhengzhou 450002, China; xianhu.liu@zzu.edu.cn

**Keywords:** porous titanium, bioactive glasses, coating, bone implant

## Abstract

Additive manufacturing enabled the fabrication of porous titanium (PT) with customized porosity and mechanical properties. However, functionalization of PT surfaces with bioactive coatings is being challenged due to sophisticated geometry and highly porous structure. In this study, a facile flow-casting technique was developed to produce homogeneous 45S5 bioactive glass (BG) coatings on the entire surface of PT. The coating weight as a function of BG concentration in a BG-PVA slurry was investigated to achieve controllable coating yield without blocking macropore structure. The annealing-treated BG coating not only exhibited compact adhesion confirmed by qualitative sonication treatment, but also enhanced the mechanical properties of PT scaffolds. Moreover, in-vitro assessments of BG-coated PT cultured with MC3T3-E1 cells was carried out having in mind their potential as bioactive bone implants. The experimental results in this study offer a simple and versatile approach for the bio-functionalization of PT and other porous biomedical devices.

## 1. Introduction

Porous design of titanium implants is gaining increasing interest recently with the development of additive manufacturing technology [1,2]. Compared to dense titanium implants, porous titanium (PT) with customized structure and porosity possesses comparable mechanical properties to natural bone to minimize the so-called “stress-shielding” effect [3,4]. In addition, the presence of interconnected macropores can facilitate the transport of nutrients and further ingrowth of bone tissue during bone regeneration [5,6]. However, Ti-based metals are intrinsically bioinert, leading to weak bone-to-implant contact and high risk of interfacial loosening in-situ [7]. Therefore, surface modification of metallic implants with bioactive coatings is of great importance to promote bone-to-implant contact in bone tissue engineering.

Bioceramics, such as bioactive glasses (BG), hydroxyapatite, and TiO_2_, are known to be used in coating form to functionalize bioactive character to inert bone scaffolds [8,9]. Various techniques have been developed to produce bioceramics coatings on dense bone implants, including line-of-sight-based processes (plasma spray [10], physical vapor deposition [11,12], etc.), electrochemical-based processes (anodic oxidation [13,14], micro-arc oxidation [15], electrophoretic deposition [16,17], etc.), and dipping-based processes [18]. In the case of porous substrates, due to their sophisticated structure, it is rather difficult to produce bioactive coatings on the entire surface area of PT without blocking the interconnected porous structure. For example, due to the unavoidable electric shielding effect of bulk titanium scaffold, it is challenging to obtain homogeneous TiO_2_ nanotube layers inside the PT by anodic oxidation [19]. Electrophoretic deposition was explored to produce bioactive coatings on PT [20] and porous graphene scaffold [21] in our previous investigation, however, the homogeneity of the coating needs to be improved since no coating was found in the core area of PT. So far, biomimetic mineralization of apatite layers [22,23], sol-gel coating [24], and dipping-based processes [18], despite their simple coating composition or time-consuming limitations, seem to be the most suitable coating techniques to create bioceramics coatings on porous substrates. A perfusion electrodeposition technique was exploited to deposit relatively thick and homogeneous calcium phosphate coatings on PT scaffold with millimeter-sized pores (1 mm pore size) [25], however, according to our preliminary investigation, the homogeneity of the deposited coating was gradually deteriorated with the decrease of pore sizes, for example, when applying PT scaffolds with a pore size of 600 μm used in this study. Therefore, new methods should be developed to efficiently deposit bioceramics or other bioactive components on PT with homogeneous morphology and tunable coating composition.

**Figure 1:**
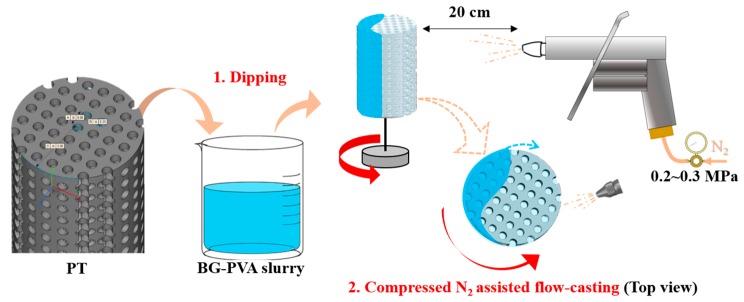
Flow-casting production of homogeneous BG coatings on PT scaffolds.

Taking advantages of room-temperature process and applicability of casting-based methods to coat porous substrates, in this study, a “flow-casting” method was developed to rapidly coat PT with BG coatings, as schematically shown in Figure 1. 3D-printed PT was firstly dipped in a BG-based slurry and then transferred to a rotation platform. With the help of moderate nitrogen gas (0.2~0.3 MPa), BG slurry was forced to flow through the macropores and gradually cast on the surface of struts in a short time, leading to the formation of a homogeneous BG coating on the entire PT surfaces without blocking the macropore structure. The key parameters of the flow-casting process were varied to obtain homogeneous BG coatings. In addition, microstructural, mechanical, and biological properties of BG-coated PT were characterized having in mind the promising potential of BG-functionalized PT in bone tissue engineering.

## 2. Experimental

### 2.1. Materials

Spherical titanium alloy powder (Ti6Al4V, TC4) with the diameter range of 15–53 μm (d50 of 43 μm) was purchased from Sino-Euro Materials Technologies of Xi’an Co., Ltd. (Xi’an, Shaanxi, China). Commercial bioactive glass powder (SCHOTT Vitryxx^®^ Bioactive Glass, 45S5 BG, Jana, Germany) with the typical composition (wt %): 45% SiO_2_, 24.5% CaO, 24.5% Na_2_O, and 6% P_2_O_5_ and particle size of 0.5~8 μm (d50 of 2.7 μm) was used. Polyvinyl alcohol powder was purchased from Sigma Aldrich (PVA, Mw of 9~10 kDa, 80% hydrolyzed, Shanghai, China). All other chemicals were supplied by Sinopharm Chemical Reagent Co., Ltd. (Shanghai, China).

### 2.2. Fabrication of PT

The PT sample was fabricated using selective laser melting (SLM) technology, which is an additive manufacturing technology for one-step production of complex geometries from raw powders. The designed geometry of PT is also shown in Figure 1. The cylindrical sample is 6 mm in diameter and 9 mm in height. The holes of 600 μm in diameter are evenly distributed in XYZ directions, and the spacing between the hole axes is 0.9 mm. Considering that the effect of pore size on stimulating osteogenesis is not fully understood, 600 μm of pore size selected in this study is mainly a model to obtain comparable porosity of PT in contrast to other relevant studies. SLM production was conducted in a Renishaw AM250 plus facility equipped with an SPI 200 W ytterbium fiber laser, operated at a wavelength of 1071 nm and a focused laser beam diameter of 75 µm (Renishaw, New Mill, UK). The samples were processed using the following optimized parameters: laser power of 200 W, hatch space of 105 μm, laser scanning speed of 1100 mm/min, layer thickness of 30 μm, and scan with stripe strategy. After the SLM process, the un-melted TC4 particles attached on the inner surfaces of struts were removed by chemical etching in an aqueous HF solution (1 vol %) for 5 min. The samples were then rinsed thoroughly with demineralized water and ethanol to remove residual HF, and finally air-dried. The porosity of the final specimen was measured to be 80 ± 3% (*n* > 30).

### 2.3. Flow-Casting Process

Aqueous PVA solution was prepared by dissolving PVA powder in hot water under continuous magnetic stirring (90 °C for 20 min). Then, different amount of BG powder were dispersed in PVA solution and subjected to alternating magnetic stirring and sonication treatments to obtain stable BG-PVA slurry. The final concentration of PVA was fixed at 15 g/L and the concentration of BG ranged from 25 g/L to 400 g/L. PVA was used to stabilize BG particles from sedimentation in suspension and then support their adhesion to titanium substrates in green coating. The as-prepared PT was immersed in BG-PVA slurry under sonication for 1 min, withdrawn, and then fixed on a rotation platform. With the help of compressed nitrogen force during rotation, the solid content in slurry was completely flow-casted on the struts in a few minutes. The coating parameters were fixed to produce homogeneous BG-PVA coatings, as marked in Figure 1. The compressed N_2_ is only used to drive the movement of BG-PVA slurry inside the PT. A moderate N_2_ pressure of 0.2~0.3 MPa was finally selected, which is not too low to trigger the movement of slurry, nor too high to blow the slurry out of PT. The BG-PVA-coated PT was dried in a desiccator at 60 °C overnight to remove completely moisture, followed by vacuum annealing treatment (1 × 10^−4^ Pa) to remove PVA binder as well as to enhance the bonding between the BG coating and PT. After a trial-and-error approach using different annealing temperatures (600~1000 °C) and time (30–120 min) aiming to achieve enhanced coating adhesion and also to avoid the mechanical deterioration of PT substrates, the best possible annealing protocol was fixed (900 °C for 1 h, heating and cooling rates of 4 °C/min). Both the uncoated and BG-coated PT were treated with the same annealing conditions before further characterizations.

### 2.4. Coating Characterizations

The coating weight as a function of BG concentration was obtained by weighting PT substrates before and after coating (analytical balance with an accuracy of 0.1 mg, *n* = 5). The bare and BG-coated cylindrical PT were cut from the middle point of the axis, and the inner surface morphology of the struts were observed by scanning electron microscope (SEM, Nova NanoSEM, FEI, Hillsboro, OR, USA). Raw and annealed BG powder using the same annealing protocol was subjected to X-ray diffraction analysis (XRD, Bruker D8, Karlsruhe, Germany, Cu Kα radiation with a step size of 0.02°) to evaluate the possible crystallization of the BG coating after annealing. According to Ryu’s method [23,26], ultrasonication tests were carried out to qualitatively evaluate the adhesion strength of annealing-treated BG coatings. The samples were ultrasonically treated (40 kHz, 250 W) in PBS for 30 min, and the reduction of coating weight was recorded to assess the adhesion (*n* = 5). It should be noted that the applied intensity of sonication was twice that used in literature. The current existing methods to quantify the adhesion strength, including scratch test, tape test, pull-off tensile test, and friction-wear test, are not suitable for coatings on porous substrates. The intensity of ultrasonication treatment used in this study is considered the highest compared to those reported in literature. Furthermore, this treatment offers qualitative evaluation of the adhesion strength on the entire surface area of PT substrates. Compression testing of the bare and BG-coated samples was carried out using an Instron 3382 tester according to ASTM E9-2009 standard. The specimen of Φ 6 × 9 mm was compressed using a lineal crosshead speed of 0.05 mm/min. The elastic modulus was calculated from the slope of the compressive stress–strain curve in the linear elastic region. At least 5 specimens were tested for each sample condition.

### 2.5. Cellular Studies

MC3T3-E1 cells were cultured in α-MEM with 10% FBS, 100 IU/mL of penicillin, and 100 μg/mL of streptomycin at 37 °C in 5% CO_2_ atmosphere. After reaching 90% confluency, cells were trypsinized using 0.25% trypsin for the in vitro experiment. MTT test was performed to evaluate cell viability on bare and BG-coated samples. The samples were autoclaved before cell culture (6 mm in diameter and 9 mm in height). The sample was transferred to a 24-well plate, and 1.5 mL of cell suspension at a density of 5 × 10^5^ cells/mL was poured on each sample and cocultured for 2 and 4 days, respectively. In this case, some cells were evenly spread on the PT scaffold and some cells settled at the bottom of the well. Considering the bare and BG-coated PT possessed a similar geometry and porosity, it was expected that the seeding efficiency on both the samples would be the same. After culture, the sample was rinsed with PBS and transferred to a new well filled with 1 mL of culture medium containing 10% of sterilized 3-(4,5)-dimethylthiahiazo(-z-y1)-3,5-diphenytetrazoliumromide (MTT) and incubated for an additional 4 h at 37 °C, allowing viable cells to metabolically reduce MTT into purple formazan. Then, 200 μL of dimethyl sulfoxide was added to each well and incubated on a shaking platform for 10 min at room temperature. The final mixture was subjected to optical density measurement (λ = 490 nm) using a multifunctional microplate reader (Synergy HT, BioTek, Winooski, VT, USA, *n* = 4).

For cell morphology observations, the samples were rinsed with PBS and fixed in 2.5% glutaraldehyde for 3 h at 4 °C after culture, and subsequently dehydrated in ethanol solutions of varying concentrations (30, 50, 70, 90, and 100%) for 15 min. The morphology of the fixed cells was then observed by SEM. The cytoskeleton organization was analyzed using Filamentous actins (F-actin) staining. After 2 and 4 days culture, cells were fixed with 4% paraformaldehyde for 15 min, permeabilized with acetone for 3 min at −20 °C, and stained with Rhodamine Phalloidin (Solarbio, Beijing, China) for 30 min and Hoechst 33258 (Solarbio, Beijing, China) for 15 min in the dark. The samples were rinsed with PBS for 3 times after each step. The stained cells were viewed under fluorescence microscopy (80i, Nikon, Tokyo, Japan, *n* = 3). It should be noted that the samples were cut from the middle point of the axis, and the inner surface of PT structs was applied for cell morphology and fluorescence observation (similar to sample preparation for SEM observation).

## 3. Results and Discussion

**Figure 2:**
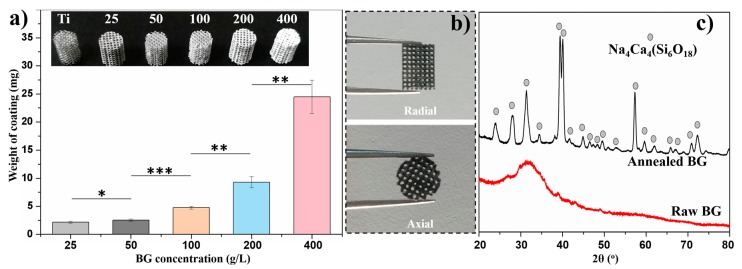
(**a**) The weight of BG-PVA coating as a function of BG concentration in slurry; the inset shows the increased white content on the coated PT with increasing BG concentration (* *p* < 0.05, ** *p* < 0.01, *** *p* < 0.001), (**b**) axial and radial observation of BG200-coated PT after annealing, (**c**) XRD patterns of raw BG and annealed BG powder using the same annealing protocol.

Solvent-casting has been well-established to produce homogeneous coatings on bulk surfaces. A low solid concentration in casting medium will yield a low deposition rate while a high solid concentration will inevitably deteriorate the homogeneity of the deposited coating due to its high viscosity. When applying PT as the coating substrate, the slurry will be retained inside the interconnected macropores (~600 μm) by capillary forces, and eventually block the macropores after drying. With the help of mild gas-flow, which is not too severe to blow out the slurry, the retained slurry is able to move through the macropores and finally the solid content could adhere homogeneously on the entire surface of the struts. The deposit weight of green BG-PVA coating as a function of BG concentration was measured as shown in Figure 2a, in which the inset showed the corresponding optical images of the BG-PVA-coated PT. The weight of the coating increased accordingly with increasing BG concentration in slurry, which was also confirmed by the increased white content on the sample. Slight variation of the coating weight when using BG concentration of less than 200 g/L indicated the reproducibility of the flow-casting method. However, a drastic increase of the coating weight was detected with a BG concentration of 400 g/L, which should be attributed to the high viscosity of BG-PVA slurry blocking partially the macropores inside PT (see inset image). Therefore, 200 g/L of BG concentration was selected to coat PT with high coating yield and interconnected macropore structure, namely BG200. As shown in Figure 2b, The BG200-coated PT after the annealing treatment was imaged from radial and axial directions, showing a completely interconnected pore structure in the presence of BG coating. The final annealing treatment was selected mainly intending to enhance the adhesion of BG coatings to PT while avoiding undesirable mechanical deterioration of PT scaffolds at excessive annealing temperatures. Meanwhile, annealing treatment will also inevitably induce crystallization of amorphous BG phase. The XRD pattern of BG powder annealed using the same protocol is shown in Figure 2c, in which raw BG powder was also measured as reference. It was found that amorphous BG particles were transformed completely into crystalline Na_4_Ca_4_(Si_6_O_18_) phase, which is in agreement with reported results in literature [27]. Although crystallization will inevitably deteriorate the bioactivity of BG, it was found by Hench et al. that the crystallized BG retained excellent bioactivity, that is, crystalline hydroxyapatite phase was detected to form on 100% crystallized BG after incubation in simulated body fluid for app. 22 h [28]. Based on these reported results, it is concluded that the produced BG coatings in this study were completely crystallized after annealing (900 °C for 1 h), and the hydroxyapatite-forming ability of BG was expected to be maintained to further improve the bioactivity of PT scaffolds.

**Figure 3:**
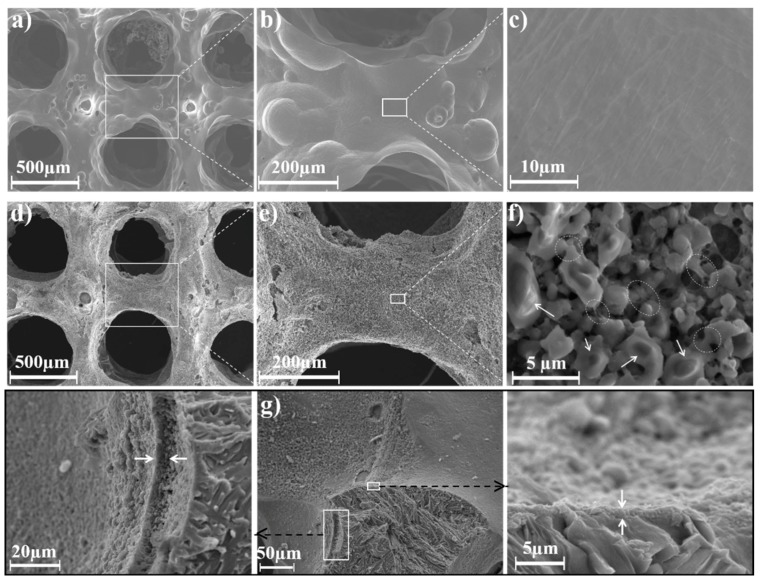
SEM images of the inner surface morphology of (**a**–**c**) bare Ti scaffold and (**d**–**f**) BG200-coated Ti scaffold, (**g**) cross-sectional images of BG200-coatings at different magnifications.

Both the bare and BG200-coated PT scaffolds were cut from the middle point of the cylinder axis, and the inner surface morphology was observed by SEM at different magnifications, as shown in Figure 3. As shown in Figure 3a, the as-produced PT exhibited ordered and interconnected macropores of about 654 ± 47 μm following the pore size of the designed model (600 μm). The high magnification images of bare PT showed a smooth surface after etching treatment. It should be noticed that acid etching treatment in this study is only intended to detach the weak-bonded titanium powders from the struts. After coating and annealing processes, as shown in Figure 3d–f, the interconnected pore structure was preserved and all the struts were coated with a crack-free and particle layer, which should be assigned to BG coatings due to the similar particle size to that of raw BG powders. No obvious decrease of macropore size was observed after BG coating, which is probably due to the negligible thickness of BG coating in contrast to the size of macropores. In addition, considering that the weight of thermal-treated BG coating is app. 6 mg compared to 300 mg of raw PT, we could conclude that the porosity of PT was not obviously changed after BG coating. It was found that BG particles were slightly melted based on the presence of sintering neck and partially melted BG particles (arrowed and circled in Figure 3f), which may improve the integrity of BG coating and its adhesion to PT substrates. Cross-sectional SEM images of the BG coating at different magnifications are shown in Figure 3g, which showed a compact connection between the BG coating and the PT strut after annealing. The thickness of the BG coating was not uniform as observed from different positions of the strut (about 6 and 2 μm as arrowed in magnified images), however, it could be observed that the entire surface of the strut was wrapped with BG coatings. In addition, the BG coating was seen to be porous due to the partial melting of BG particles. Indeed, higher annealing temperatures were previously employed to further densify the BG coating, however, such densification process was found to lead to cracks and even detachment of the BG coating from the Ti matrix. Considering the significantly different thermal expansion coefficient (TEC) between Ti (9.6 × 10^−6^ °C^−1^) [29] and bioactive glasses (15.1 × 10^−6^ °C^−1^) [30], tensile stresses would be generated during the cooling process. In contrast, with proper design of annealing protocol, a porous structure of BG coating could minimize TEC mismatching between BG coating and Ti matrix, which is favorable to avoid cracking and maintain the tight adhesion of annealed BG coatings.

**Figure 4:**
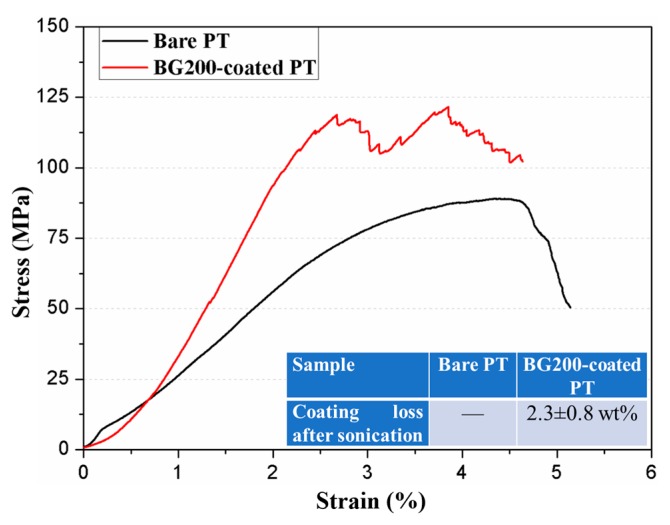
Characteristic stress−strain curves of the bare and BG200-coated PT during compression test (displacement of 0.05 mm/min); the inset table shows the coating loss after sonication treatments to evaluate the adhesion strength.

Characteristic stress–strain curves of bare and BG200-coated PT during compression tests are shown in Figure 4. The stress fluctuations during the yield stage of BG200-coated PT, with respect to the smooth curve of bare PT, should be assigned to the fracture of BG coatings from the struts. In addition, the strain ratio of PT was significantly reduced from 4.5% to 2.5% in the presence of a rigid BG coating, which is also an evidence of bonding between BG coating and Ti substrates. The adhesion strength of the BG coating was qualitatively evaluated by sonication of BG200-coated PT in PBS for 30 min (40 kHz, 250 W), which led to only 2.3 ± 0.8% of weight loss after sonication, as introduced in the inset table of Figure 4. Yu et al. [23] reported that mineralized apatite coating was severely detached from PT substrates during sonication, while in our cases, the thermal-treated BG200 coating remained stable after double-intensity sonication. The excellent adhesion of BG coating on PT scaffolds could be assigned to the partial oxidation of metal substrates and diffusion of metal atoms from Ti substrates into the BG coating during annealing [31]. In addition, according to our previous experience on room-temperature fabrication of BG-polymer coatings [17,32,33], the green BG coatings could be retained on metallic substrates with desirable adhesion and a slow degradation rate. Therefore, we expect that the annealed BG coatings on PT scaffolds should be more stable in body fluid, which will be analyzed in following biological assessments.

The corresponding elastic modulus of bare and BG200-coated PT was calculated directly from stress–strain curves, which are 2.71 ± 0.14 GPa and 3.33 ± 0.20 GPa, respectively, as shown in Table 1. With the help of the compact bonding between BG coating and PT substrates, it is suggested that the compression stress could be partially transferred to rigid BG coating, leading to slightly increased elastic modulus of BG-coated PT scaffolds. As also listed in Table 1, various techniques were developed to fabricate PT scaffolds with tailored porosity and minimized elastic modulus, such as 3D printing, wire entanglement, and powder metallurgy techniques. Powder metallurgy is usually used to fabricate PT scaffolds with relatively low porosities. Compared to wire-entangled PT scaffolds with comparable porosity, 3D-printed PT based on either EBM or SLM principle (applied in this study) exhibited significantly higher elastic modulus. The increased porosity of PT scaffolds was found to induce a drastic decrease of elastic modulus in all cases. However, with the incorporation of biological factors, for example, gelatin fillers [34] or BG coatings in our study, the elastic modulus could be further strengthened. The reported elastic modulus values of cancellous bone and cortical bone are 0.01~3 [35] and 3~30 [36,37] GPa, respectively. The calculated elastic modulus values of bare and BG200-coated PT are close to the range of both cancellous bone and cortical bone. It is expected that with proper design of PT as well as the deposit yield of BG coatings, the composition and mechanical properties of BG-coated PT scaffolds could be further adjusted to meet their needs as either cancellous or cortical bone implants.

**Figure 5:**
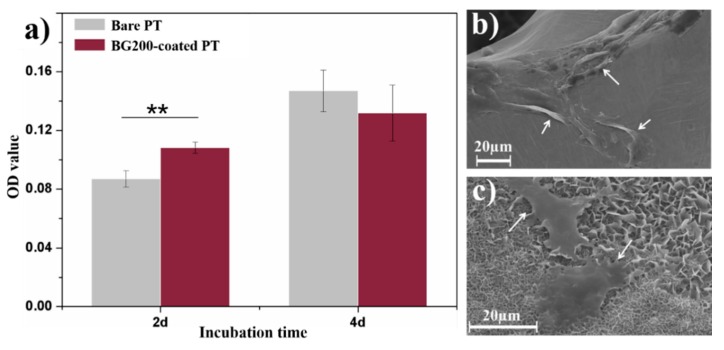
(**a**) MTT test of cells cultured on bare and BG200-coated PT for 2 and 4 days (** *p* < 0.01), and the morphology of cells cultured on (**b**) bare and (**c**) BG200-coated PT for 4 days.

Viability of cells cultured on bare and BG200-coated PT for 2 and 4 days were comparatively studied by means of MTT test as shown in Figure 5a. Cells on the coated sample exhibited significantly higher viability (*p* < 0.01) than that on bare PT at day 2, while comparable cell viability was detected on both samples at day 4. The morphology of cells cultured on both samples for 4 days is shown in Figure 5b,c. Cells adhered on BG200-coated PT present tabular morphology rather than aggregating into elongated clusters on bare PT, indicating relatively higher osteoblast compatibility of BG200-coated PT compared to bare PT. Furthermore, it was found in Figure 5c that the BG coating was completely covered by a flake-like layer, which should be assigned to the crystalline hydroxyapatite formed during mineralization of BG phase [32].

**Figure 6:**
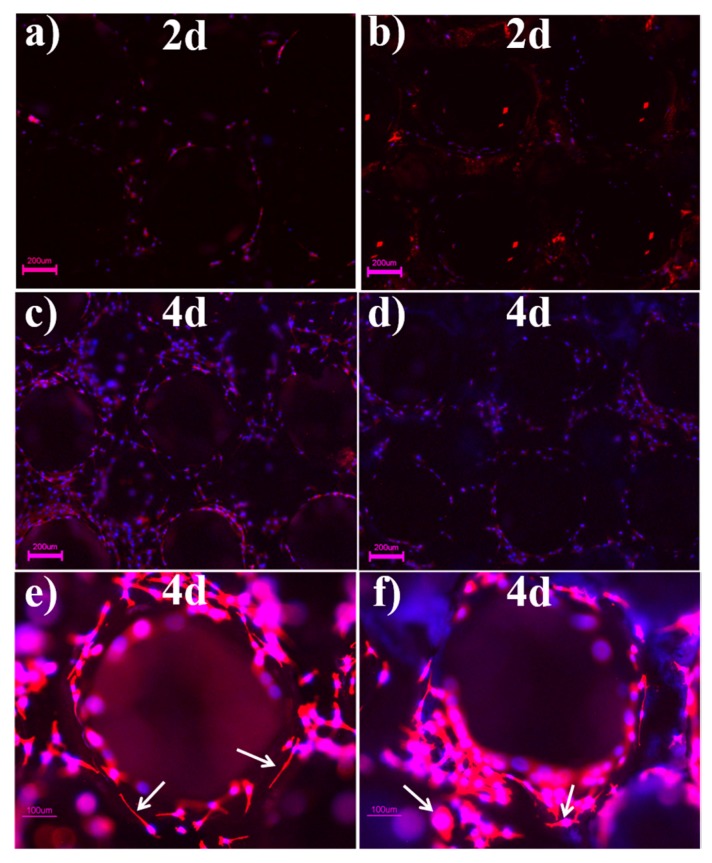
Fluorescent images of cells cultured on bare (**a**,**c**,**e**) and BG200-coated (**b**,**d**,**f**) Ti scaffolds after 2 and 4 days, respectively. Scale bars in (**a**–**d**) represent 200 μm and scale bars in (**e**–**f**) represent 100 μm.

Fluorescent images of cells cultured on bare and BG200-coated PT for 2 and 4 days are shown in Figure 6, where the cytoskeleton and nuclei are stained in red and blue, respectively. Cells were homogeneously distributed on both samples, indicating an excellent cytocompatibility of two types of samples. Relatively more cells (red content) were observed on BG200-coated PT at day 2 while a comparable number of cells was found on bare and BG200-coated PT at day 4, which is in agreement with MTT results in Figure 5a. Moreover, it was observed from the high magnification images that the cells on bare PT presented elongated morphology compared to the cells on BG-coated PT presenting tabular morphology at day 4 (Figure 6e–f). BG coatings have been reported to not only enhance osteoblastic proliferation, but also induce osteogenic differentiation [32,41]. In addition, it was reported that human mesenchymal stem cells (hMSCs) with elongated morphology exhibited lowered osteogenesis compared to polygonal-like hMSCs, which is positively correlated with down-expression of cell morphological indexes including integrin β1 and N-cadherin [42]. Therefore, the tabular morphology of MC3T3-E1 cells on BG200-coated PT, with respect to the elongated cell morphology on bare PT, may indicate the occurrence of early osteogenic differentiation at day 4. Considering the well-established osteogenic function of BG material in clinical therapy [43], we expect that the BG-coated PT is likely to promote osteogenic activity in future assessments.

## 4. Conclusions

A facile flow-casting method was developed to produce homogeneous BG coatings on PT substrates without blocking the interconnected macropores. The coating yield could be tailored by varying BG concentration in the range of 25~200 g/L. In addition, BG200-coated PT exhibited excellent coating adhesion and strengthened elastic modulus of 3.33 ± 0.2 GPa after annealing treatment, which is promising as candidates of bone implants. Although the annealed BG coating exhibited nonuniform thickness varying from 2 μm to 6 μm, in-vitro biological evaluation confirmed an improved osteoblast activity, probably owing to the excellent bioactivity of the BG phase. Summarizing, flow-casting of BG coatings, possibly combined with other bioactive components or functional molecules, suggested a simple and effective approach for the biofunctionalization of PT or other porous devices, which will be advantageous for designing optimal scaffolds for biomedical application.

## Figures and Tables

**Table 1:** Typical processing methods of PT scaffolds and the corresponding porosity and elastic modulus values in comparison with our results.

Material	Processing Method	Porosity (vol %)	Elastic Modulus (GPa)	Ref.
Bare PT	Selective laser melting (SLM)	80 ± 3	2.71 ± 0.14	Our work
BG200-coated PT	SLM + annealed BG coating		3.33 ± 0.20	Our work
Bare PT	Ti-wire entanglement	44.7	1.05	[38]
Bare PT	Electron beam melting (EBM)	60	5.48 ± 0.54	[39]
		67	3.85 ± 0.56	
		75	2.23 ± 0.68	
Bare PT	Powder metallurgy	30	44.2 ± 0.6	[40]
		40	24.7 ± 2.5	
		50	15.4 ± 1.1	
PT filled with gelatin gel	Ti-wire entanglement	50	7.4	[34]
		60	3.9	
		70	2.5	
